# New insight into the mechanism underlying the silk gland biological process by knocking out fibroin heavy chain in the silkworm

**DOI:** 10.1186/s12864-018-4602-4

**Published:** 2018-03-26

**Authors:** Yong Cui, Yanan Zhu, Yongjian Lin, Lei Chen, Qili Feng, Wen Wang, Hui Xiang

**Affiliations:** 1 Guangzhou Key Laboratory of Insect Development Regulation and Application Research, Institute of Insect Science and Technology, School of Life Sciences, South China Normal University, Guangzhou, 510631 China; 2 Center for Ecological and Environmental Sciences, Northwestern Polytechnical University, Xi’an, 710129 China

**Keywords:** *Bombyx mori*, CRISPR/Cas9, Transcriptome, *Bmfib-H*, Proteasome, Autophagy

## Abstract

**Background:**

Exploring whether and how mutation of silk protein contributes to subsequent re-allocation of nitrogen, and impacts on the timing of silk gland degradation, is important to understand silk gland biology. Rapid development and wide application of genome editing approach in the silkworm provide us an opportunity to address these issues.

**Results:**

Using CRISPR/Cas9 system, we successfully performed genome editing of *Bmfib-H*. The loss-of-function mutations caused naked pupa and thin cocoon mutant phenotypes. Compared with the wild type, the posterior silk gland of mutant showed obviously degraded into fragments in advance of programmed cell death of silk gland cells. Comparative transcriptomic analyses of silk gland at the fourth day of the fifth instar larval stage(L5D4)identified 1456 differential expressed genes (DEGs) between posterior silk gland (PSG) and mid silk gland (MSG) and 1388 DEGs between the mutant and the wild type. Hierarchical clustering of all the DEGs indicated a remarkable down-regulated and an up-regulated gene clade in the mutant silk glands, respectively. Down-regulated genes were overrepresented in the pathways involved in cancer, DNA replication and cell proliferation. Intriguingly, up-regulated DEGs are significantly enriched in the proteasome. By further comparison on the transcriptome of MSG and PSG between the wild type and the mutant, we consistently observed that up-regulated DEGs in the mutant PSG were enriched in protein degrading activity and proteasome. Meantime, we observed a series of up-regulated genes involved in autophagy. Since these protein degradation processes would be normally occur after the spinning time, the results suggesting that these progresses were activated remarkably ahead of schedule in the mutant.

**Conclusions:**

Accumulation of abnormal fib-H protein might arouse the activation of proteasomes as well as autophagy process, to promote the rapid degradation of such abnormal proteins and the silk gland cells. Our study therefore proposes a subsequent process of protein and partial cellular degradation caused by mutation of silk protein, which might be helpful for understanding its impact of the silk gland biological process, and further exploration the re-allocation of nitrogen in the silkworm.

**Electronic supplementary material:**

The online version of this article (10.1186/s12864-018-4602-4) contains supplementary material, which is available to authorized users.

## Background

The domestic silkworm (*Bombyx mori*) is a significant economic insect for synthesizing and spinning cocoon, which is an important material for not only textiles and industrial applications but also biomaterials and cosmetics [1, 2]. The main components of cocoon comprise fibroins [75% (*w*/w)] and sericins [25% (w/w)]. Silk fibroin which chiefly consists of the fibroin heavy chain (Fib-H), fibroin light chain (Fib-L) and the 25-kD polypeptide proteins (fibrohexamerin protein/P25), at a molar ratio of 6:6:1 [3], is synthesized in the posterior silk gland (PSG), accumulates and coats with sericin when it goes through the middle silk gland (MSG), and is then secreted via the anterior silk gland (ASG) [4].

The character of cocoon of silkworm is a typical complex trait and the expression of silk protein gene is strictly subject to time and space constraints. In general, by the last larval instar, the gland cells no longer divide but undergo chromosomal endoreduplication. As a result, a large amount of DNA replication results in 200,000 to 400,000 fold increase in DNA content [5]. The silk glands grow sharply and synthesize silk proteins dramatically [6]. Once the cocoon is formed, the silk glands degenerate during the larva-pupa transition [7]. Apoptosis, autophagy and cathepsins play essential roles in this process and autophagy precedes apoptosis [8, 9].

There have been extensive efforts in exploring the regulatory mechanisms of silk protein synthesis. For example, several transcription factors related to the transcriptional regulation of fibroin in silkworm have been identified, including silk grand factor-1(SGF-1), SGF-2,SGF-3,fibroin-modulator-binding protein-1, SGF-4,*Bmsage* and *Bmdimm* [10–15]. On the other hand, there are studies based on mutant and, trying to elaborate the mechanism of silk production. For example, early studies on naked pupa and thin cocoon mutant indicated that mutations in the either Fib-H or Fib-L genes resulted in the reduction of silk production, suggesting that both Fib-H and Fib-L are necessary for the secretion of silk fibroin [16, 17]. However, these studies are still preliminary or failed to demonstrate the molecular regulatory network on silk synthesis. Recently, Zhong et al. [18, 19] used transcriptomic and proteomic approaches had analyzed the another mutant, i.e. ZB strain which had significant low silk production, and found that the efficiency of silk protein expression in ribosomes was reduced and protein degradation was more active in the ZB strain. They suggested the decreased silk protein expression and the enhanced silk protein degradation are the major factors in lower silk production. However, since causative mutation of ZB strain is unknown, the key factor and the related pathway that caused this mutation phenotype are still unclear. As is known to all, silk protein is the end product of nitrogen metabolism at the end of the larval stage of silkworm. Loss-of-function of silk protein could result in excess nitrogen accumulated in other tissues, such as, fat body [20]. Therefore, we suspected that the mutation of silk protein may evoke subsequent re-allocation of nitrogen. Today, there is no illumination of these possible progresses, either its biological impact on silk gland development.

Benefitting from the rapid development and the wide application of genome editing technologies, especially the newly developed clustered regularly interspaced short palindromic repeats/RNA-guided Cas9 nucleases (CRISPR/Cas9) system has been successfully used in silkworms and proven to be an efficient genome editing tool for exploring gene functions [21–23]. We are now able to address the above issue, by generating loss-of-function mutant of silk protein and further exploring the morphological and molecular changes occurred in silk glands. In the current work, using CRISPR/Cas9 system, we performed genome editing of *Bmfib-H* gene in *B. mori* and found that loss-of-function mutations of fib-H caused severe thinner and lighter cocoons accompanied with premature autophagy. Comparative transcriptomic analyses of MSG and PSG between 4-day-old fifth instar larvae (L5D4) individuals of wild type and the mutant indicate that protein processing and transportation may be weakened, but autophagy are obviously activated in the PSG of mutant, which is remarkably ahead of the programmed cell death (PCD) in the normal progress of silkworm development. In general, this study provides a significant clue for us to understand the molecular mechanism of silk synthesis and silk gland biological process in the silkworm.

## Results and discussion

### Designing on sgRNA targets and detection of mutation types of *Bmfib-H*

**Fig. 1:**
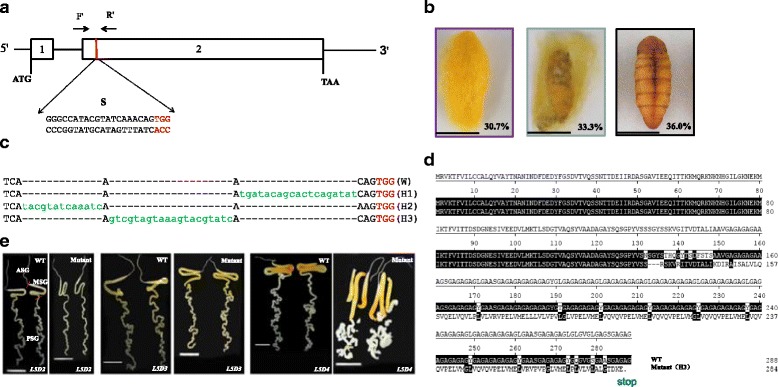
Cas9/sgRNA mediated gene editing of *fib-H* of the silkworm. **a** Schematic diagram of the sgRNA-targeting sites. The two boxes indicate the two exons of *Bmfib-H*, and the black line indicates the gene locus. The sgRNA targeting sites S is located on the sense strand of exon-2. F′ and R’ were used to anneal the upstream and downstream regions of the targeting site. The sgRNA targeting sequence is in black, and the protospacer adjacent motif (PAM) sequence is in red. **b** The different phenotypes of Cas9/sgRNA induced mutations. The left is the normal cocoon, the middle side is the thin-layered cocoon and the right is the naked pupae. **c** Various types of insertion mutations screened from homozygous mutant silkworms (G3). H1, 19 bp insertion; H2, 13 bp insertion, and H3, 20 bp insertion. Deletions are indicated by hyphens and insertions are shown in green lowercase letters. The PAM sequence is in red. **d** Comparison of predicted amino acid sequences of H3 and wild type amino acid sequence. The missing amino acids are replaced with dashes. The numbers on the right indicate the amino acid residue positions of the proteins. Premature stop codons are shown in blue letters and black point. **e** Silk glands of the wild type (WT) and the mutant from the 2nd day to 4th day of 5th larvae stage. All the scale bars represent 1 cm

Fib-H is a considerably large protein rich in glycine, serine, alanine and tyrosine [20]. And the genomic structure of *Bmfib-H* is composed of two exons and one intron. After screening for sgRNA sites over the exons of *Bmfib-H* (see methods), we identified a 23-bp sgRNA target site located on exon 2 (Fig. 1a). SgRNA that was synthesized in vitro were mixed with Cas9 mRNA and injected into preblastoderm embryos of silkworm *Nistari* strain. At G0 generation, we totally obtained 75 larvae that survived to the pupae or larvae-pupae, about one third of them presented thin cocoon layer, another one third were naked pupae or arrested during the pupation and nearly all of these mutants died during this course (Fig. 1b). Since the mutants of naked pupae were unable to develop into adult moth, we further randomly selected larvae-sloughs of 30 individuals with the thin-cocoon phenotype and conducted genotyping with PCR products sequencing. As shown in Additional file 1 Figure S1, the multiple peaks in chromatograms of PCR-product sequencing at the region flanking the target position were indicative of mosaic mutation. We therefore identified several individuals with such mosaic mutation genotypes and pair-mating to produce G1 generation offspring populations. We maintained G1 generation and selected 7 thin-cocoon individuals for pair-mating again to produce G2 generation. Totally G3 generations of selection were made until homozygous individuals were obtained. In the G3 generation, we found three mutation types (H1, 19 bp insertion; H2, 13 bp insertion and H3, 20 bp insertion), which resulted in the frame shift mutation and premature stop at 153aa, 137aa and 284aa, respectively (Fig. 1c and d). Furthermore, we chose one type of mutants (H3) for homozygosis mutant population maintaining and at G4 generation, we conducted morphological comparison of their silk gland with the wild type during the fifth instar. As shown in Fig. 1e, on the second and third day of the fifth larval instar, morphology of silk gland were nearly the same between the wild type and the mutant. However, on the fourth day, the mutant posterior silk gland degraded into small fragments. Such degradation phenomenon should not occur in the L5D4 in the normal development processes of the silkworm (Fig. 1e). Normally, the silk glands begin to disintegrate at the prepupal stage, which is 2 days after the wandering stage [24].

### Identification of DEGs from silk gland comparative transcriptomic analysis

**Fig. 2:**
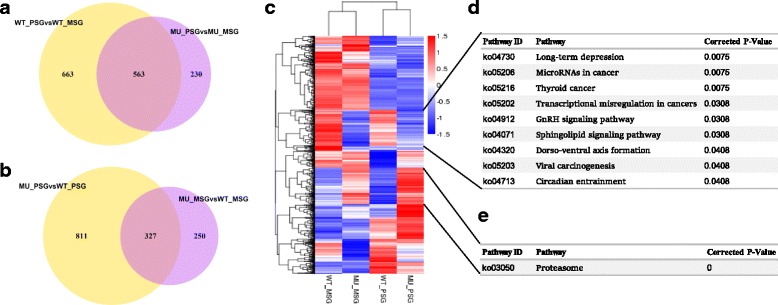
Expression patterns of the differentially expressed genes in the MSG and PSG of the wild type and the mutant. **a** Venn diagram of the differential expressed genes (DEGs) between the mid silk gland and posterior silk gland (**b**) Venn diagram of the DEGs between the wild type and the mutant in the same tissue. MSG, middle silk gland; PSG, posterior silk gland. **c** Heatmap and Hierarchical clustering of the differentially expressed genes. **d** KEGG enrichment pathways for genes that are nearly uniformly down-regulated in both the MSG and PSG of the mutant. **e** KEGG enrichment pathways for genes that are generally obviously upregulated in both the MSG and PSG of the mutant. Corrected *p*-value: *p*-value in hypergeometric test after correction. All pathways had Corrected *p*-value < 0.05

RNA-seq of MSG and PSG from wild type and mutant at L5D4, yielded 6.61 ~ 8.56 Gb clean data (Additional file 2 Table S1). Using these data we identified 1534 novel transcirpts (Additional file 3 Table S2). By comparative transcirptomic analyses based on the Fragments Per kb Per Million Reads (FPKM) (Additional file 4 Table S3) [25], we totally identified 1456 tissue differential expressed genes (DEG) (see Additional file 5 Table S4) between PSG and MSG among which 1226 are from wild type and 793 are from the mutant. Meantime, we identified 1388 DEGs between the mutant and the wild type among which 577 are in MSG and 1138 are in PSG (Fig. 2a and b). Notably, as to DEGs between MSG and PSG, we clearly observed more of these DGEs specifically expressed in the wild type (663) than in the mutant (230) (Fig. 2a). Therefore we focused further functional annotation on these 663 genes, and found that they are enriched in “primary active transmembrane transporter” and “catalytic activity”. Consistently, KEGG analysis showed that, they are enriched in the pathway of “protein processing in endoplasmic reticulum” (Additional file 6 Table S5). Transmembrane transport was involved primarily in proteins for the transport of some specific substances, such as transporter for inorganic ions, sugar, synaptic vesicular amine and protein [18] “Protein processing in endoplasmic reticulum” is a significant posttranslational proteins modification, which is essential to protein production and directly related to the transport and secretion of proteins [26, 27]. Fibroin was the largest and most abundant silk protein produced by silkworms [21]. Normally, the processing and transport of proteins in the endoplasmic reticulum (ER) of silk glands are highly differentiated in PSG and MSG [24, 28]. However, in the mutant, these differences in protein metabolism might be weakened. In accordance with these results, we speculated that the specialization of the posterior silk gland was reduced after knockout fibroin heavy chain in the silkworm.

We further generated heatmap of all the above DEGs followed by hierarchical clustering based on their average level of expression of the four replicates separately. Interestingly, there are two remarkable clades in the heatmap. One includes genes that are nearly uniformly down-regulated in both the MSG and PSG of the mutant (totally 425 genes), and the other is on the contrary, generally obviously upregulated (377 genes), especially in the mutant PSGs (Fig. 2c). Enrichment analyses indicated that the down-regulated genes are overrepresented in the pathways involved in “MicroRNAs in cancer”, “Thyroid in cancer” and “Transcriptional misregulation in cancer” (Fig. 2d). Genes in these pathways are functionally important in cell cycle regulation, DNA replication and cell proliferation. This result suggests an overall repressed activity of endoreplication as well as cell cycle in the silk gland cells of the mutant, which might account for their drastically small silk gland. On the other hand, it is intriguing that the 377 up-regulated DEGs are significantly enriched in the only pathway, i.e. “proteasome” strongly (Fig. 2e), suggesting that the silk proteins of the mutant are subject to severe degradation, which is consistent to the morphological feature of the mutant silk gland, as we observed in the L5D4.

### Comparative silk gland transcriptome indicates conspicuous activation of protein degradation in the PSG of the mutant

Based on our observation of morphology silk gland between the mutant and the wild type, there are sharp differences in the PSG, whereas in the MSG, the differences are somewhat mild. Consistently, PSG specific DEGs are over three times than those specific in MSG (Fig. 2b). Furthermore, DEGs in MSG were not enriched in any GO terms or KEGG pathways. These results suggested that knocking out *Bmfib-H* may have little effect on the middle silk gland. However, for DEGs in PSG, we found up-regulated genes in the mutant were enriched in such cellular component as the proteasome complex, proteasome core complex and alpha-subunit complex. As to molecular function, they were enriched in threonine-type endopeptidase and threonine-type peptidase activity. For biological process, these up-regulated genes were enriched in protein catabolic process, cellular protein catabolic process, proteolysis involved in cellular protein catabolic process, cellular macromolecule catabolic process and macromolecule catabolic process (Additional file 7 Table S6). Results of KEGG analyses of these up-regulated genes indicated an enriched pathway, i.e. proteasome (Additional file 7 Table S6), as discussed in the part of GO enrichment analysis.

**Fig. 3:**
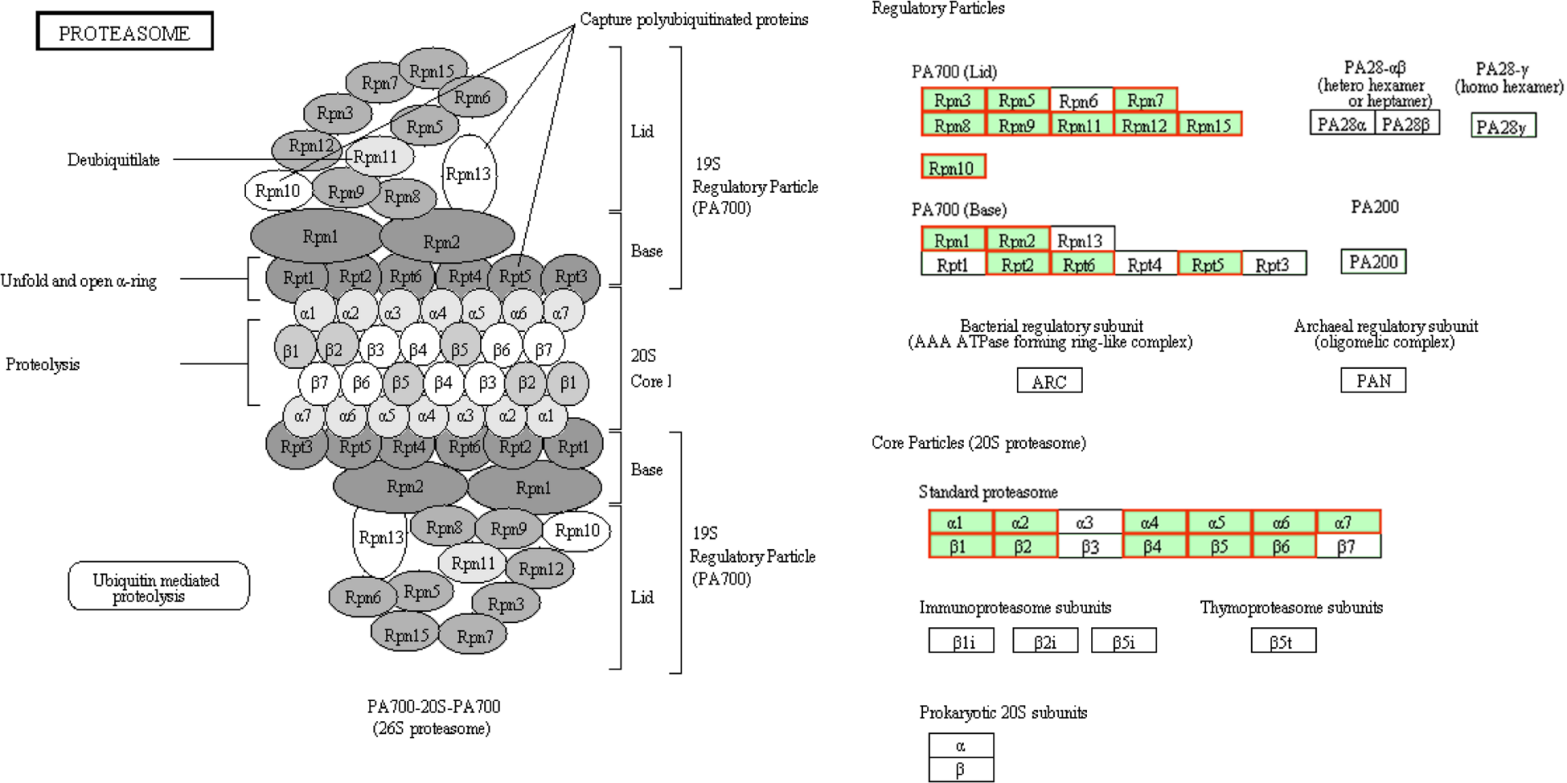
Scheme of proteasome pathway. The up-regulated transcripts in the *Bmfib-H* gene knock-out PSG are indicated by a red frame

The silk gland degradation is accomplished via PCD processes during the larval-pupal metamorphosis [9, 24]. The ubiquitin-proteasome pathway (UPP) plays important role in the PCD of the silk gland [24]. Here in this study, at L5D4, a time point that fibroin should have been produced normally and plentifully, we whereas observed obvious highly activated proteasome pathway in the posterior silk gland of mutant. Proteasome is a large protein complex comprising a 20S proteolytic core (CP), in which proteins are digested to short peptides, and two 19S regulatory particle (RP), responsible for substrate recognition and transport into the core particle, which are composed of 14 and 19 different subunits, respectively [29], and together form the 26S structure [30–37]. The 26S proteasome serve as a quality-control system that rapidly degrades abnormally folded proteins or damaged proteins whose accumulation would interfere with normal cell function and viability [38–40]. Here all of these up-regulated genes in this pathway encode protein members of the proteasome (Fig. 3). For example, *BGIBMGA002953* encodes 26S proteasome non-ATPase regulatory subunit 14, also known as 26S proteasome non-ATPase subunit Rpn11, which is one of the 19 fundamental subunits of a complete assembled 19S proteasome complex [29]. *BGIBMGA003009* was thought to encode proteasome subunit beta type-7, which might play role in the ATP/ubiquitin-dependent nonlysosomal protein degradation [41]. *BGIBMGA013898* encodes proteasome subunit alpha type-5. This protein is one of the 17 essential subunits that contribute to the complete assembly of 20S proteasome complex [42].

When abnormal proteins accumulate to exceed the cell’s degradative capacity, cells activate the heat shock proteins (hsps) response [39, 43, 44] and induce the synthesis of more proteasomes to promote the rapid degradation of such abnormal proteins [45]. Here in this study, since CRISPR/Cas9 mediated knockout of *Bmfib-H* factually resulted in shift-frame mutation occurred at 132 amino acid (aa) and premature stop codon at 284aa of the fib-H (Fig. 1d), whose expression is still derived by the strong promoter of *Bmfib-H*. Although the expression level of *Bmfib-H* transcript in the mutant (FPKM = 95,208) was relatively lower than that in the wild type (FPKM = 397,078), the transcripts of *Bmfib-H* were extremely abundant in the mutants (FPKM = 95,208), with the 2nd highest expression level among all the transcribed coding genes (Additional file 8 Table S7). Therefore, it is sensible to propose that these extremely abundant transcripts were further translated. Accordingly, these extremely abundant transcripts were further translated to the nonsense proteins with 284aa in length, resulting in accumulation of the abnormal proteins, which could accounts for the activation of synthesis for more proteasomes, as we have observed. Supporting to this inference, we found that the three important heat shock proteins (hsp90, hsp40 and hsp23) in the silkworm are significantly upregulated in the PSG of the mutant (Additional file 9: Figure S2).

**Fig. 4:**
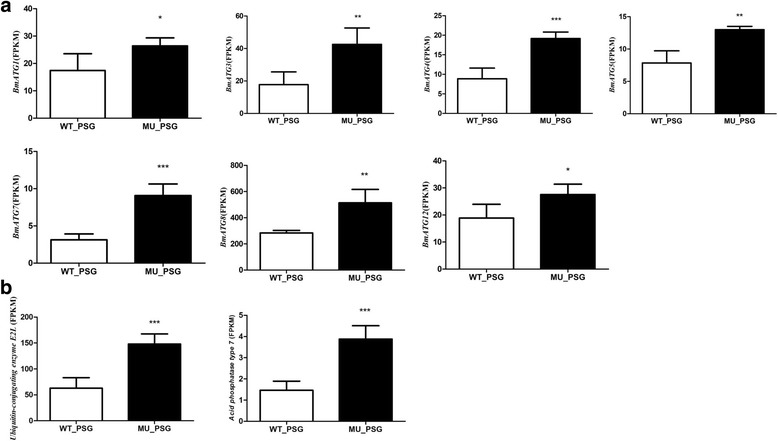
Expression levels of up-regulated DEGs related to protein degradation. **a** FPKM of the autophagy-related genes (ATG). **b** FPKM of the two important genes involved in autophagy and proteasome pathway. Ubiquitin-conjugating enzyme E2L functions in targeting a protein for degradation in the proteasome complex. Acid phosphatase is an indirect autophagic marker. *, *p* < 0.05; **, *p* < 0.01; ***, *p* < 0.001

Degradation via proteasome requires mark of unneeded protein firstly, by another kind of protein, i.e. ubiquitin, a small regulatory protein found in most tissues of eukaryotic organisms. During this process, ubiquitin-conjugating enzyme E2L performs the second step in the ubiquitination reaction that targets a protein for degradation. As expected, ubiquitin-conjugating enzyme E2L was significantly up-regulated in mutant PSG (Fig. 4).

Alternative to proteasome, Eukaryotic cells have another avenues for protein degradation called autophagy [46], which could be also induced by ubiquitin. Unlike the proteasome system, autophagy mainly degrades long-lived cytosolic proteins and organelles. The degradation of larval organs or tissues during metamorphosis is a general character among Lepidoptera, and the timing of this degradation is strictly controlled [9]. In addition, autophagy occurs at a basal level under normal conditions, but could be dramatic induced by changes of environmental conditions [46]. In this study, when knock-out of *Bmfib-H* gene, we found the posterior silk glands (PSG) of the mutant degraded into many small fragments at L5D4, compared with wild-type, indicating the beginning of these long-lived proteins degradation. Supporting to our speculation, some autophagy-related genes (ATG) were up-regulated in the mutant PSG (Fig. 4), such as i) *BmATG1*, which is the sole serine/threonine protein kinase [47] and is essential for autophagosome biogenesis, ii) *BmATG8*, that is characterized as a microtubule-associated protein required for autophagy [48], and iii) *BmATG7,* a protein is required to form a ubiquitin-like conjugation system which is involved in the biogenesis of autophagic vesicles [49, 50]. Besides to these ATGs, we also observed up-regulation of an indirect autophagic marker [9], the gene encoding acid phosphatase (*BGIBMGA008293*) (Fig. 4).

Normally, the silk gland of *B. mori* grows slowly during the early stage, rapidly increases in weight and size during the fifth larval instar, and once the cocoon has been formed, begins to degenerate [7]. However, in the fib-H deficient mutant, the process of degradation in the PSG obviously happened in advance of PCD. It is interesting that these degradation processes seem not involve apoptosis, an important process in PCD. There were no apoptosis-related genes were significantly up-regulated in the mutant PSG. We speculated that the apoptosis may occur later than the autophagy and apoptosis intervenes only when the posterior silk gland volume is drastically reduced and, once activated, PSG completely disappears [9]. In this study, the posterior silk gland of mutant still exists at L5D4, just degraded into many small fragments.

## Conclusions

In this work, we generated *B. mori* mutants by targeted editing of *Bmfib-H* gene using CRISPR/Cas9 system. Knock-out of *Bmfib-H* gene in the silkworm resulted in an exceedingly thin cocoon, naked pupae or arrested metamorphosis during the larvae-pupae transition stages. The 20-bp insertion in the Fib-H resulted in deficient mutation of this gene by introducing premature and frame-shift protein of *Bmfib-H*. Silk gland transcriptome comparisons between the mutant and the wild type revealed that DEGs associated with proteasome, threonine-type endopeptidase activity and autophagy were up-regulated in the posterior silk glands of mutant. The results strongly suggest that accumulation of abnormal or mutant proteins in the mutant PSG might lead to more activation of proteasomes synthesis as well as autophagy process to promote the rapid degradation of such abnormal proteins and the silk gland cells, possibly by an unknown mechanism to activate ubiquitin to mark these abnormal proteins. Our study therefore proposes a subsequent process of protein and partial cellular degradation caused by mutation of silk protein, which might be helpful for understanding its biological impact on silk gland development, and further exploration on the re-allocation of nitrogen in the silkworm.

## Methods

### Silkworm strains

A multivoltine, silkworm strain, Nistari, was used for all experiments. Larvae were reared on fresh mulberry leaves at 25 °C and relative humidity of 70%–80% under standard conditions [51].

### In vitro transcription of Cas9 mRNA and sgRNA

The designed sgRNA sites follow the GGN19GG rule [52–54]. Target sequences with high and low GC content which might affect targeting cleavage activity were left out [55]. We identified one 23 bp sgRNA targeting sites at exon II of *Bmfib-H* (Fig. 1a). The sgRNA DNA template was synthesized by PCR, with Q5® High-Fidelity DNA Polymerase (NEB, America), one oligonucleotide (*Bmfib-H*-sgF1) that encoded the T7 polymerase binding site, sgRNA targeting sequence and overlapsequence were separately annealed to a common oligonucleotide that encoded the remainder of the sgRNA sequence (sgRNA-R) [55] (Additional file 10 Table S8). The reaction conditions were as follows: 98 °C for 2 min, 35 cycles of 98 °C for 10 s, 60 °C for 30 s, and 72 °C for30s, followed by a final extension period of 72 °C for forever.

The sgRNA were synthesized based on the DNA template in vitro with MAXIscript® T7 Kit (Ambion, Austin, TX, USA) according to the manufacturer’s instruction. The Cas9 gene template used in this work was provided by the Shanghai Institute of Plant Physiology and Ecology (Shanghai, China). Cas9 mRNA was prepared using the mMESSAGE mMACHINE® T7 kit (Ambion, Austin, TX, USA) according to the manufacturer’s instruction.

### Micro-injection of Cas9/sgRNA

A total of 630 fertilized eggs were collected and injected within 6 h after oviposition. A mixture of Cas9 mRNA (1000 ng /μL) and sgRNA (1000 ng /μL) were mixed and injected into the preblastoderm Nistari embryos (about 12 nl / egg) using a micro-injector (FemtoJet®, Germany), according to the standard protocol. And the injected eggs were incubated at 25 °C for 9–10 days until hatching. Among them, 79 eggs (12.5%) hatched. For the unhatched eggs, we found that the proportion of mutant individual is rather high (100%, data not shown).

### Selection and maintenance of mutant populations

The above hatched individuals were G0 individuals. After phenotypic screening on G0 individuals, we randomly selected larvae-sloughs of 30 individuals with thin-cocoon phenotypes to extract genomic DNA separately for genotyping by sequencing PCR products. As shown in Additional file 1 Fig. S1, the multiple peaks in chromatograms of PCR-product sequencing at the region flanking the target position were indicative of mosaic mutation. We thus identified several individuals with such mosaic mutation genotypes and pair-mating to produce G1 generation offspring populations. Due to factual feeding situation (in winter), we maintained G1 generation with small population size and selected 7 thin-cocoon individuals for pair-mating to produce G2 generation.

At G2 generation, a large number of thin-cocoon and naked-pupa phenotypes appeared. By genotyping on a batch of these mutants (genomic DNA extracted from the sloughs), we were able to select the moths that have the same shift-frame mutation genotypes to pair-mate and produced G3 generation.

At G3 generation, individuals that had obviously thin-cocoon phenotypes were selected for genotyping. At this generation, we successfully identified homozygous mutants with shift-frame mutation. There were three kinds of homozygous mutation (H1, H2 and H3). We then selected the moths with homozygous and same shift mutation genotypes for pair-mating and produced G4 generation. We chose one type of mutants (H3) for homozygosis mutant population maintaining.

### Genomic DNA extraction and mutagenesis analysis

Genomic PCR, followed by sequencing, was carried out to identify the *Bmfib-H* mutant alleles induced by the CRISPR/Cas9 system. Genomic DNA was extracted by TIANamp Blood DNA Kit (Tiangen Biotech, Beijing) according to the manufacturer’s instruction. The PCR conditions were as follows: 94 °C for 2 min, 35 cycles of 94 °C for 30 s, 57 °C for 45 s, and 72 °C for 30 s, followed by a final extension period of 72 °C for 10 min. The amplified fragments were cloned into a pMD™19-T Simple Vector (Takara, Japan). The primers that were designed to detect mutagenesis in targeted sites were as follows: Primer-F′ and Primer-R’ detected targeting sites (Additional file 10 Table S8).

### RNA-seq

Four duplicate (two male and two female) samples were set for the wild type and the mutant (H3), respectively. For each sample, middle and posterior silk gland were dissected respectively, stored in dry ice and then sent to Novogene company for RNA extraction and RNA-seq. The Sequencing libraries were generated using NEBNext® Ultra™ RNA Library Prep Kit for Illumina® (NEB, USA) following manufacturer’s recommendations. The library preparations were sequenced on the Illumina HiSeq™ 2000 platform and 150 bp paired-end reads were generated.

Sequenced raw data were qualified, filtered, built, and mapped to the reference silkworm genome database (http://silkworm.genomics.org.cn/) by and TopHat v2.0.12 [56]. HTSeq v0.6.1 was used to count the reads numbers mapped to each gene. And then FPKM of each gene was calculated based on the length of the gene and reads count mapped to this gene [57]. Novel transcirpts were identified by Cufflinks v2.1.1 [58].

### Identification of differential expressed genes and functional enrichment analyses

To analysis the differentially expressed genes (DEGs), the DESeq R package 1.12.0 (TNLIST, Beijing, China) was used with to identify DEGs with Corrected *p*-value < 0.05. For further analysis, Gene Ontology (GO) enrichment analysis of differentially expressed genes was implemented by the GOseq R package, in which gene length bias was corrected. GO terms with corrected *P* value less than 0.05 were considered significantly enriched among the DEGs. After getting GO enrichment analysis, we used KOBAS software to test the statistical enrichment of differential expression genes in KEGG pathways.

### Availability of supporting data

Reads of transcriptome were deposited in the Sequence Read Archive (SRA) database of NCBI with the accession number SRP131538 (https://www.ncbi.nlm.nih.gov/sra/SRP131538).

## Additional files

Additional file1: **Figure S1.** The multiple peaks near the target declared the mutation happened. Target site is underlined. PAM sequence is bold in red. (DOCX 16 kb)

Additional file 2: **Table S1.** Summary of the RNA-Seq data. (PDF 31 kb)

Additional file 3: **Table S2.** Gene structure annotations of the novel transcripts. (DOCX 20 kb)

Additional file 4: **Table S3.** Expression levels (FPKM) of all the annotated genes. (XLS 1181 kb)

Additional file 5: **Table S4.** Information of the differentially expressed genes. (XLS 5797 kb)

Additional file 6: **Table S5.** GO enrichment and KEGG enrichment analysis of unique DEGs between middle- (MSG) and posterior-(PSG) silk gland specifically in the wild type. (XLSX 262 kb)

Additional file 7: **Table S6.** GO enrichment and KEGG enrichment analysis of up-regulated DEGs in the posterior silk grand between the wild type and the mutant. \ (DOCX 17 kb)

Additional file 8: **Table S7.** The top10 expressed genes in the mutant posterior silk gland. (DOCX 17 kb)

Additional file 9: **Figure S2.** Expression levels of the three heat shock proteins up-regulated in the posterior silk gland of the mutant. (XLS 18 kb)

Additional file 10: **Table S8.** Primers used in this study. (PDF 58 kb)

## Abbreviations

ASG: Anterior silk gland
ATG: Autophagy-related gene
CP: Proteolytic core
CRISPR/Cas9: Clustered regularly interspaced short palindromic repeats/RNA-guided Cas9 nucleases
ER: Endoplasmic reticulum
FPKM: Fragments Per kb Per Million Reads
GO: Gene Ontology
Hsp: Heat shock protein
KEGG: Kyoto Encyclopedia of Genes and Genomes
L5D4: The fourth day of the fifth instar larval stage
L5D6: The sixth day of the fifth instar larval stage
MSG: Middle silk gland
PCD: Programmed cell death
PSG: posterior silk gland
RP: Regulatory particle
UPP: Ubiquitin-proteasome pathway
