# Supplementary material for: New insight into the mechanism underlying the silk gland biological process by knocking out fibroin heavy chain in the silkworm
Source: BMC Genomics. 2018 Mar 26;19:215. doi: 10.1186/s12864-018-4602-4 (PMC5870212; doi:10.1186/s12864-018-4602-4)
Supplement: Supplementary file 1 — Figure S1. The multiple peaks near the target declared the mutation happened. Target site is underlined. PAM sequence is bold in red. (DOCX 16 kb) [file 12864_2018_4602_MOESM10_ESM.docx]

| **Additional file 10 Table S8 Primers used in this study.** | | |
| --- | --- | --- |
| **Primer name** | **Primer sequence (5’–3’)** | **Primer purpose** |
| *Bmfib-H*-sgF1 | TAATACGACTCACTATAGGGCCATACGTATCAAACAGGTTTTAGAGCTAGAAATAGCAAGTTAAAATAAGGCTAGTCC | Preparation of sgRNA templates |
| sgRNA-R | AAAAGCACCGACTCGGTGCCACTTTTTCAAGTTGATAACGGACTAGCCTTATTTTAACTTGCTATTTCTAGCTCTAAAA | Preparation of sgRNA templates |
| Primer-F’ | TTCCGACGGTAACGAGTCCA | Identification of somatic mutations |
| Primer-R’ | CTACTCCTTGTCCGTACCCAG | Identification of somatic mutations |
