# Supplementary figures and images for: New insight into the mechanism underlying the silk gland biological process by knocking out fibroin heavy chain in the silkworm
Source: BMC Genomics. 2018 Mar 26;19:215. doi: 10.1186/s12864-018-4602-4 (PMC5870212; doi:10.1186/s12864-018-4602-4)

GGGCCATACGTATCAAACAG**TGG**

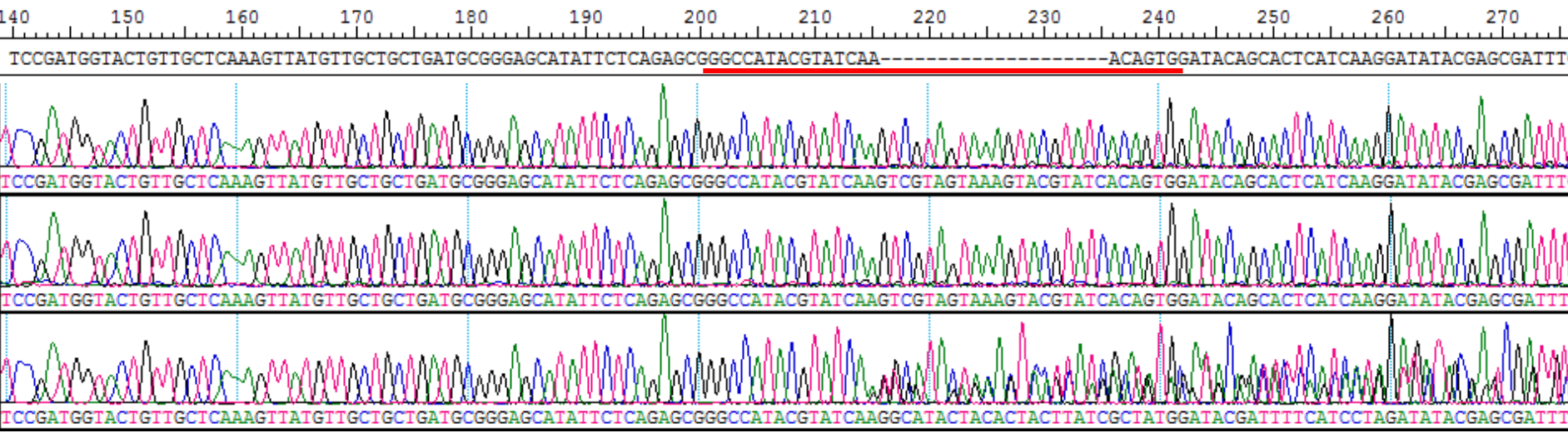

Supplement: Supplementary file 2 — Table S1. Summary of the RNA-Seq data. (PDF 31 kb) [file 12864_2018_4602_MOESM1_ESM.pdf]

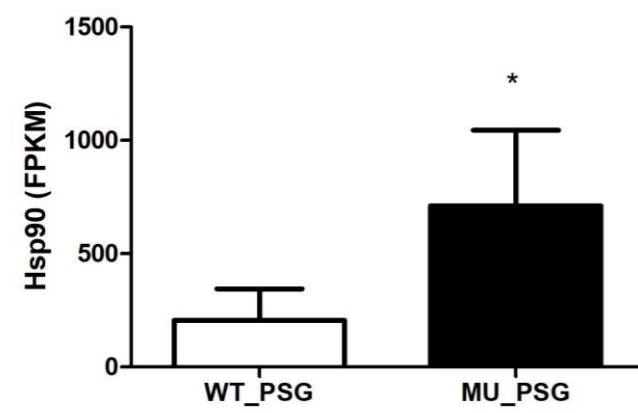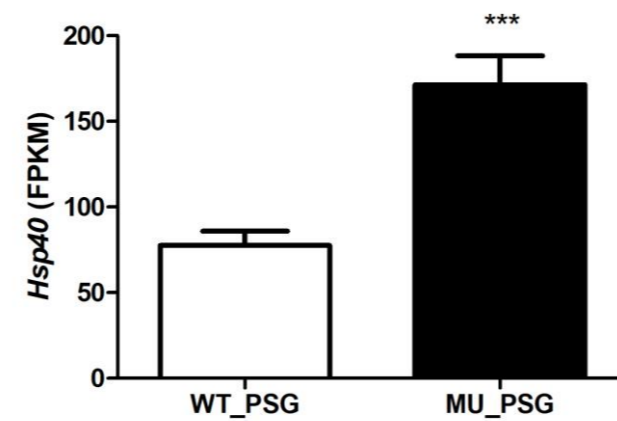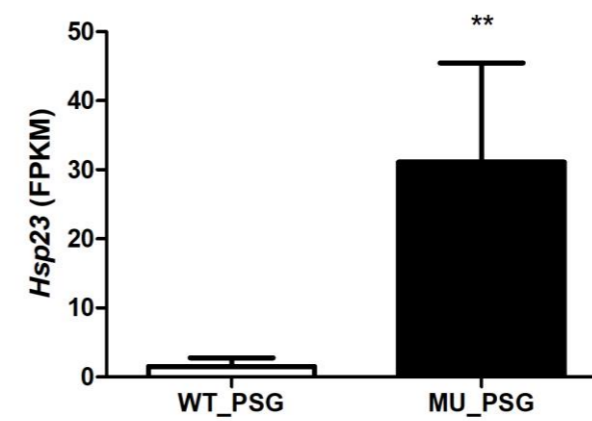

Supplement: Supplementary file 10 — Table S8. Primers used in this study. (PDF 58 kb) [file 12864_2018_4602_MOESM9_ESM.pdf]
