# Supplementary material for: New insight into the mechanism underlying the silk gland biological process by knocking out fibroin heavy chain in the silkworm
Source: BMC Genomics. 2018 Mar 26;19:215. doi: 10.1186/s12864-018-4602-4 (PMC5870212; doi:10.1186/s12864-018-4602-4)
Supplement: Supplementary file 3 — Table S2. Gene structure annotations of the novel transcripts. (DOCX 20 kb) [file 12864_2018_4602_MOESM2_ESM.docx]

**Additional file 2: Table S1 Summary of the RNA-Seq data.**

| **Sample name** | | **Raw reads** | | **Clean reads** | | **Clean bases (Gb)** | | **Q30 (%)** | |
| --- | --- | --- | --- | --- | --- | --- | --- | --- | --- |
| WT_MSG1M | | 56,718,832 | | 54,855,122 | | 8.23 | | 92.49 | |
| WT_MSG2M | | 55,122,832 | | 53,258,228 | | 7.99 | | 92.17 | |
| WT_MSG3F | | 56,393,588 | | 53,921,594 | | 8.09 | | 91.39 | |
| WT_MSG4F | | 48,935,408 | | 47,034,334 | | 7.06 | | 91.76 | |
| MU_MSG1M | | 52,071,094 | | 50,297,034 | | 7.54 | | 92.45 | |
| MU_MSG2M | | 57,968,746 | | 55,847,828 | | 8.38 | | 91.96 | |
| MU_MSG3F | | 57,210,232 | | 53,813,020 | | 8.07 | | 90.88 | |
| MU_MSG4F | | 46,501,384 | | 45,121,056 | | 6.77 | | 92.89 | |
| WT_PSG1M | | 57,595,924 | | 54,487,526 | | 8.17 | | 86.95 | |
| WT_PSG2M | | 56,131,036 | | 53,206,176 | | 7.98 | | 87.47 | |
| WT_PSG3F | | 60,830,388 | | 57,037,408 | | 8.56 | | 85.08 | |
| WT_PSG4F | | 55,181,924 | | 51,705,738 | | 7.76 | | 86.99 | |
| MU_PSG1M | | 54,002,434 | | 51,345,592 | | 7.70 | | 93.20 | |
| MU_PSG2M | | 52,372,474 | | 50,012,906 | | 7.50 | | 92.76 | |
| MU_PSG3F | | 46,212,916 | | 44,095,750 | | 6.61 | | 92.89 | |
| MU_PSG4F | | 54,551,176 | | 51,955,456 | | 7.79 | | 93.03 | |

WT: wild type; MU: mutant; MSG: mid silk gland; PSG: posterior silk gland; M: male; F: female.

Gb: Giga base; Q30: percentage of bases with a Phred value of at least 30.
