# Supplementary material for: New insight into the mechanism underlying the silk gland biological process by knocking out fibroin heavy chain in the silkworm
Source: BMC Genomics. 2018 Mar 26;19:215. doi: 10.1186/s12864-018-4602-4 (PMC5870212; doi:10.1186/s12864-018-4602-4)
Supplement: Supplementary file 7 — Table S6. GO enrichment and KEGG enrichment analysis of up-regulated DEGs in the posterior silk grand between the wild type and the mutant. \ (DOCX 17 kb) [file 12864_2018_4602_MOESM6_ESM.docx]

| **Additional file 6 Table S5 GO enrichment and KEGG enrichment analysis of unique DEGs between middle- (MSG) and posterior-(PSG) silk gland specifically in the wild type** | | |
| --- | --- | --- |
| **ID** | **Description** | **Corrected p-Value** |
|  | *Terms from the function ontology* |  |
| GO:0015399 | Primary active transmembrane transporter activity | 0.0130 |
| GO:0003824 | Catalytic activity | 0.0260 |
| ko04141 | Protein processing in endoplasmic reticulum | 0.0047 |
| Corrected p-value: p-value in hypergeometric test after correction. | | |
