# Supplementary material for: New insight into the mechanism underlying the silk gland biological process by knocking out fibroin heavy chain in the silkworm
Source: BMC Genomics. 2018 Mar 26;19:215. doi: 10.1186/s12864-018-4602-4 (PMC5870212; doi:10.1186/s12864-018-4602-4)
Supplement: Supplementary file 8 — Table S7. The top10 expressed genes in the mutant posterior silk gland. (DOCX 17 kb) [file 12864_2018_4602_MOESM7_ESM.docx]

| **Additional file 7 Table S6 GO enrichment and KEGG enrichment analysis of up-regulated DEGs in the posterior silk grand between the wild type and the mutants** | | |
| --- | --- | --- |
| **ID** | **Description** | **Corrected p-Value** |
|  | *Terms from the component ontology* |  |
| GO:0000502 | Proteasome complex | 4.44E-12 |
| GO:0005839  GO:0019773 | Proteasome core complex  Proteasome core complex, alpha-subunit complex | 1.31E-10  2.65E-06 |
|  | *Terms from the function ontology* |  |
| GO:0004298 | Threonine-type endopeptidase activity | 2.07E-11 |
| GO:0070003 | Threonine-type peptidase activity | 2.07E-11 |
|  | *Terms from the process ontology* |  |
| GO:0030163 | Protein catabolic process | 0 |
| GO:0044257 | Cellular protein catabolic process | 0 |
| GO:0051603 | Proteolysis involved in cellular protein catabolic process | 0 |
| GO:0044265 | Cellular macromolecule catabolic process | 0.01 |
| GO:0009057 | Macromolecule catabolic process | 0.01 |
| ko03050 | Proteasome | 1.56E-19 |
| Corrected p-value: p-value in hypergeometric test after correction. | | |
|  |  |  |
